# Huge hematoma following extracorporeal shock wave lithotripsy leading to nephrectomy

**DOI:** 10.1016/j.eucr.2025.103164

**Published:** 2025-08-22

**Authors:** Mehdi Dadpour, Jamal Sadr, Seyed Mohammad Hashem Montazeri, Mina Vishteh, Sina Poorsalimi, Hamidreza Samadpour

**Affiliations:** a Shahid Labbafinejad Medical Center, The Center of Excellence in Urology, Urology and Nephrology Research Center, Research Institute for Urology and Nephrology, Shahid Beheshti University of Medical Sciences, Tehran, Iran; b Shahid Labbafinejad Medical Center, Shahid Beheshti University of Medical Sciences, Tehran, Iran

**Keywords:** ESWL, Nephrectomy, Hematoma, Extracorporeal shockwave lithotripsy

## Abstract

Extracorporeal shockwave lithotripsy (ESWL) is a noninvasive and relatively safe method for treating small urinary tract stones, however it may be accompanied with some important complications including hematoma. In this report, we review an instructive and rare case of a patient with massive renal hematoma following ESWL. Despite full conservative management, due to patient instability, emergent exploration and nephrectomy was inevitable. Massive hematoma leading to nephrectomy following ESWL procedure is very rare, but urologists should be aware. Also, if a patient is unstable and don't response to conservative treatment, nephrectomy may be considered after primary resuscitation.

## 1 Introduction

Extracorporeal shockwave lithotripsy (ESWL) is a noninvasive and relatively safe method for treating urinary tract stones, in which the stones are broken into smaller, more mobile pieces with the help of shock waves. This elective treatment method reduces the financial burden of urolithiasis on the treatment system by reducing the need to use invasive methods.^1^

Despite all of the advantages, this method also has complications in less than one percent of patients, like hematomas, which are usually self-limiting with supportive treatments and follow-up.^2^^,^^3^

In this report, we review an instructive and rare case of a patient with massive renal hematoma that led to nephrectomy in a patient undergoing extracorporeal lithotripsy.

## 2 Case presentation

A 61-year-old man presented to the emergency department two days before the start of the Nowruz holiday-the annual 13-day festival that marks the beginning of spring in Iran-with complaints of left flank pain and hematuria. The patient also had a history of extracorporeal lithotripsy for the treatment of kidney stones the day before his presentation. He had no medical history other than controlled Hypertension. Also, he had only used acetaminophen after ESWL as a painkiller.

**Fig. 1:**
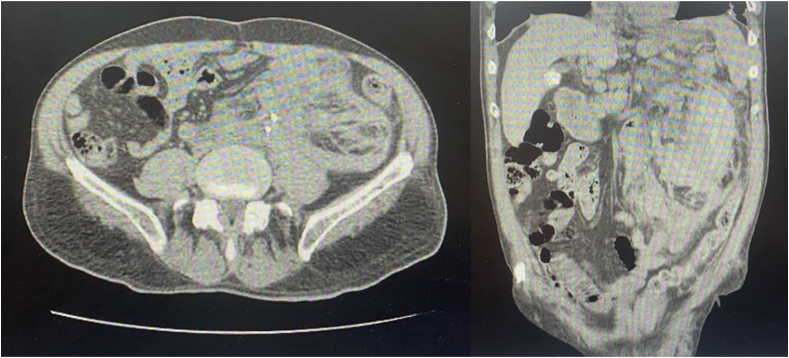
Transverse and coronal sections of the CT scan show a massive hematoma of the left kidney.

On examination, his vital signs were recorded as follows: The blood pressure was 110 on 70 mmHg, pulse rate was 110 beats per minute, respiratory rate was 18 cycles per minute, and O2 saturation was 94–97 %. In the first CBC test, the Hemoglobin (Hb) was 8.5 g/dl, and the Platelet count was 220 billion/L. Also, the creatinine was raised to 2.5 mg/dl, and the INR ratio was 1.1, so the patient became complete bed rest and resuscitated with fluid and electrolytes were carried out. The patient underwent Abdominopelvic CT scan to evaluate the presence of visible injuries, and, as a result, a massive left retroperitoneal hematoma was found (Fig. 1). two units of iso group packed cells were transfused to the patient.

The patient was admitted to the intensive care unit (ICU). Despite two units of pack cell transfusion, The hemoglobin level dropped to 8 g/dl, the platelet dropped to 120 billion/L, and the creatinine level raised to 2.6 mg/dl the day after ICU admission. He received two more packed cell units with two FFP units (fresh frozen plasma). Still, the hemoglobin level dropped to 6.6g/dl, the platelet level dropped to 84 billion/L, and the creatinine level increased to 2.8 mg/dl on the second day after admission.

**Fig. 2:**
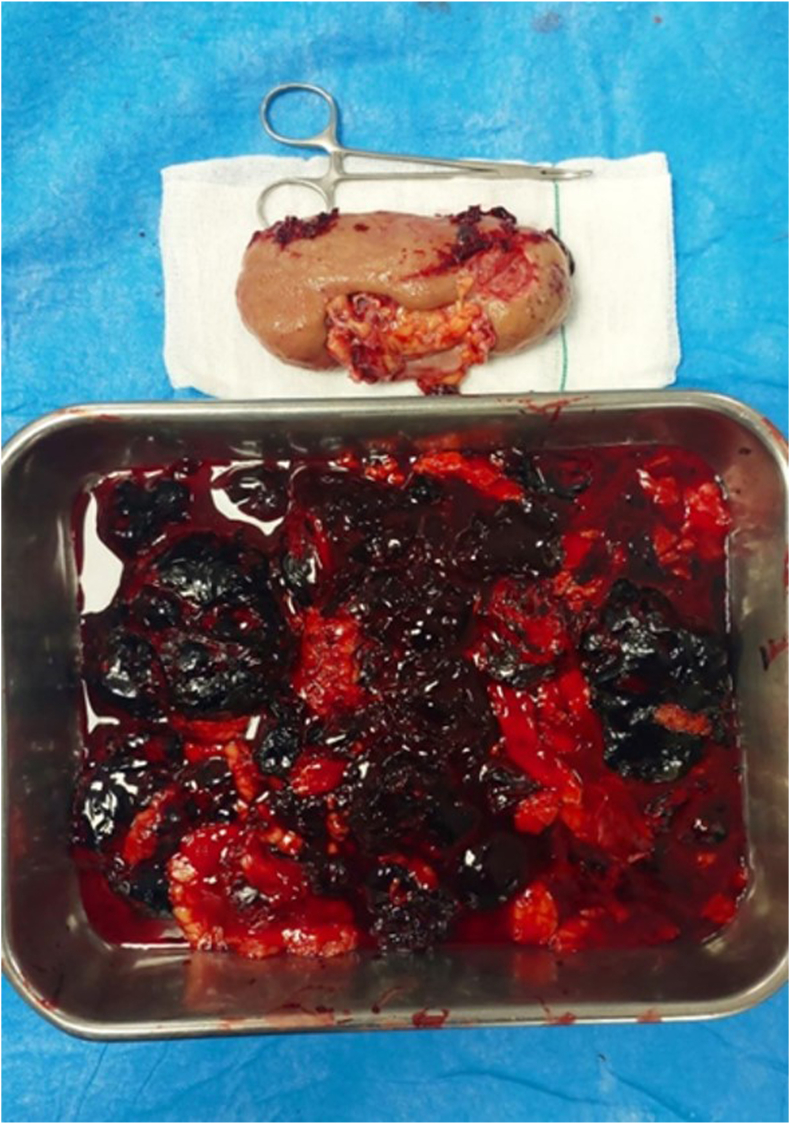
The kidney removed during nephrectomy surgery with hematoma and damaged tissues.

Despite full conservative management, patient's vital sign became unstable gradually: blood pressure dropped to 80/50 mmhg, PR increased to 125/min, so he considered a candidate for emergent exploration. He was transferred to the operating room, after general anesthesia the patient was positioned in flank position, a flank incision between 11th and 12th ribs was made to explore the retroperitoneal space. During the exploration high amount of retroperitoneal hematoma due to shattered kidney were found (Fig. 2). There was no active bleeding but due to the patient history and severe renal injury, nephrectomy was inevitable. Two more units of pack cell, two units of FFP and cryoprecipitates were transfused during surgery.

In the post op period, patient's condition was improved, vital signs remained stable, no hemoglobin drop was found. No significant drain leakage was observed. Creatinine level decreased to 1.6mg/dl. No more blood product was needed. The bowel movement returned on POD 2. He was discharged from the hospital on POD 4. two weeks follow up was uneventful.

## 3 Discussion

The patient mentioned in this report is a very rare case who developed renal hematoma with decreased haemoglobin, thrombocytopenia, and increased creatinine following ESWL, which has no response to conservative treatments and final saved his life by nephrectomy surgery.

Although ESWL is considered safe, its side effects should be considered in less than one percent of the patients, which have been studied in three categories: infectious, related to urinary flow obstruction and related to tissue damages. hematomas can be mentioned as tissue damage complication of ESWL, which are self-limiting usually and followed up until they are absorbed and reduced, but our patient's complication progressed differently than usual.^2^

Lee et al. evaluated 6177 patients with 10,887 periods of ESWLs during ten years and found that the incidence rate of post-ESWL hematoma is 0.32 %. They mentioned that all of these cases were managed conservatively. Hypertension, higher body mass index, and large stone size were the risk factors for post-ESWL hematoma.^4^ In a study of 10,953 patients, only 31 cases of hematoma were reported, and Hypertension was identified as an essential risk factor for the occurrence of this complication^5^ also, diabetes can increase the risk of complications.^6^

Strategies have also been proposed to reduce tissue injury following ESWL, such as discontinuing anticoagulant medications, controlling hypertension and diabetes, and initiating lithotripsy with low-frequency pulses to allow time for renal vasoconstriction.^3^ Perhaps the high number of patients visiting the ESWL clinic just two days before a long holiday has caused the use of pulses with a short time interval and high energy, which also affected the occurrence of complications for our patients. However, Our claim cannot be proven.^7^ Proper patient selection including careful preoperative imaging evaluation to assess stone burden and anatomical anomalies and control the risk factors like high blood pressure before the procedure would be necessary.

Jdaini et al. reported a 60-year-old man who underwent ESWL. He had severe abdominal pain 4 h after ESWL. CT scan showed a subcapsular hematoma. The patient was discharged after conservative treatment for two days.^8^

Para et al.^9^ reported a 48-year-old male with massive retroperitoneal hematoma after ESWL without any comorbidity or coagulopathy. The patient was managed conservatively. In terms of performing a nephrectomy for controlling hematoma, our case was sporadic because there are few reports about performing this procedure in such cases. Adequate resuscitation with fluids and blood products before surgery seemed appropriate.

Inoue et al.^10^ reported an old patient with a massive haemorrhagic right kidney after ESWL that the patient died with uncontrolled bleeding despite a nephrectomy.

ESWL is generally known to have few complications, but by studying related cases, it seems necessary to pay attention to risk factors, possible complications, timely diagnosis and treatment. to the best of our knowledge, we did not find any cases in the literature where the mentioned treatment result in a hematoma requiring a nephrectomy that saved the patient from death.

## 4 Conclusion

Massive hematoma leading to nephrectomy following ESWL procedure is very rare, but urologists should be aware. Also, if a patient is unstable and don't response to conservative treatment, nephrectomy may be considered after primary resuscitation.

## CRediT authorship contribution statement

**Mehdi Dadpour:** Writing – review & editing, Writing – original draft, Supervision, Project administration, Methodology, Investigation, Data curation, Conceptualization. **Jamal Sadr:** Writing – review & editing, Writing – original draft, Visualization, Validation, Conceptualization. **Seyed Mohammad Hashem Montazeri:** Writing – review & editing, Writing – original draft, Visualization, Validation. **Mina Vishteh:** Visualization, Validation, Investigation, Conceptualization. **Sina Poorsalimi:** Investigation. **Hamidreza Samadpour:** Visualization, Validation, Data curation.
